# 537 Efficacy and Safety of Tranexamic Acid in Burn Surgery: A Comprehensive Systematic Review

**DOI:** 10.1093/jbcr/iraf019.166

**Published:** 2025-04-01

**Authors:** Kyle Mangum, Jaina Eckert, Osaid Alser, Dan Stuart, Bryce Stash

**Affiliations:** Texas Tech University Health Sciences Center School of Medicine; Texas Tech University Health Sciences Center School of Medicine; Texas Tech University Health Science Center, Department of Surgery; Texas Tech University Health Science Center; Texas Tech University Health Science Center

## Abstract

**Introduction:**

Tranexamic acid (TXA) is an antifibrinolytic agent primarily used to prevent bleeding. In recent years, multiple studies have investigated its use in burn surgery. While some systematic reviews and meta-analyses have been conducted, none have comprehensively reviewed all available literature on this topic in a way that effectively translates to clinical application. A thorough and well-organized review is needed to clarify current findings on this complex issue.

**Methods:**

We conducted a systematic review following PRISMA guidelines, focusing on TXA, burn surgery, and blood loss. A reproducible search was executed in August 2023 and repeated in 2024 across multiple major databases including PubMed, Ovid MEDLINE, and Embase. Three independent authors screened studies for inclusion, and a narrative review was performed on selected articles.

**Results:**

Fourteen articles were included: three randomized controlled trials (RCTs), one prospective observational study, two retrospective observational studies (ROS), four case reports, and four conference presentations. Studies were categorized into three main groups: intravenous (IV) TXA, topical TXA, and TXA in high-risk populations. IV TXA showed significant reductions in blood loss per affected area in two RCTs, reduced packed red blood cells (pRBC) transfusion in one RCT and two ROS, and reduced colloid transfusion in one RCT and one ROS. The studies on topical TXA were too heterogeneous for effective comparison. In high-risk subpopulations, TXA was beneficial as part of a multimodal strategy to minimize blood loss. Across all categories, TXA exhibited no significant adverse complications, mortality, or increased length of stay.

**Conclusions:**

Current data provide moderate support for TXA use in burn surgery, showing benefits in reducing pRBC and colloid transfusions and blood loss per affected area, with minimal safety concerns. IV TXA should be used in patients at higher risk of bleeding prior to burn surgery. Topical TXA use should be considered a safe alternative option for local hemostasis. Future research should focus on the cost-effectiveness of TXA versus blood products and its impact on inflammation and healing, which could facilitate wider adoption in burn care.

**Applicability of Research to Practice:**

TXA presents potential benefits with low risk. This review identifies gaps in the literature and suggests directions for future studies.

**Funding for the Study:**

N/A

